# Dyeing Performance of Disperse Dyes on Polyester Fabrics Using Eco-Friendly Carrier and Their Antioxidant and Anticancer Activities

**DOI:** 10.3390/ijerph16234603

**Published:** 2019-11-20

**Authors:** Alya M. Al-Etaibi, Morsy Ahmed El-Apasery

**Affiliations:** 1 Natural Science Department, College of Health Science, Public Authority for Applied Education and Training, Fayha 72853, Kuwait; 2 Dyeing, Printing and Textile Auxiliaries Department, Textile Industries Research Division, National Research Centre, 33 El Buhouth St., Dokki, Cairo 12622, Egypt; elapaserym@yahoo.com

**Keywords:** eco-friendly carrier, polyester fabric, fastness properties, dyeing performance

## Abstract

Both non eco- and eco-friendly carriers were utilized for accelerating the dyeing rate of polyethylene terephthalate fabrics (PET) dyed with disperse dyes at 100 °C. Fastness properties of the dyed fabrics showed good and excellent results. Finally, the prepared disperse dyes 1 and 2 showed potent anti-tumor cytotoxic activity in vitro using MCF-7 cells (human breast cancer cell line), HepG-2 cells (human Hepatocellular carcinoma), HCT-116 (colon carcinoma), A-549 cells (Lung carcinoma cell line), and anti-oxidant activities.

## 1. Introduction

Disperse dyes are generally non-ionic synthetics with saving dissolvability in water that can hold similarly and better substantively for hydrophobic fibers, for example, nylon and polyester [1,2,3,4,5,6].

To acquire adequate dyeability, the dyeing of polyester fabrics must be performed at high temperature and high pressure or by utilizing a carrier, which suggests huge energy utilization and ecological tainting [7].

The carrier is an organic compound that quickens dyeing by separating or dissolving dye aggregates and carrying them to the fiber–water interface in little amounts that are enough to be absorbed by the material. An eco-friendly carrier must lower both the volatility and odor, be free of chlorinated aromatics, and meet ecological standards [8,9,10,11]. Recently, pyridone and fused heterocyclic pyridone derivatives and their analogs are well known in the fields of chemotherapy and medicinal chemistry due to their anticancer, antimetabolite, antiviral, and antitumor activities [12,13]. In continuation of our study aimed to synthesize pyridone disperse dyes that exhibited biological activity [14], we studied the dyeing behavior of pyridone disperse dyes in the presence of eco- and no eco-friendly carriers at 100 °C. It should be noted that the dyeing behavior of the dyes under investigation is expressed in K/S values (K is a measure of light absorption and S is a measure of light scattering). Additionally, the antioxidant and anticancer activities of these pyridone disperse dyes were evaluated.

## 2. Materials and Methods 

### 2.1. Low Temperature Dyeing

Samples of 100% polyester fabric were put into a beaker containing dye shade 2% of disperse dye 1 or dye 2 in the presence of Matexil DA-N (supplied by ICI Company, London, UK) as a dispersing agent, Tanavol EP 2007 (supplied by TANATEX Chemicals B.V., Ede, Gelderland, the Netherlands), or commercial HC as carriers at 100 °C dyeing temperatures and the dyeing time of 1 h. First, the dye was dissolved in 10 drops of dimethylformamide as a solvent and mixed with Matexil DA-N, then water was added in a 1:50 liquor ratio and the pH was adjusted to 4.5. After the dyeing process, the dyed samples were subjected to reduction clearing with 1 g/L of sodium hydrosulfite and 1 g/L of sodium hydroxide for 10 min at 60 °C. 

### 2.2. Measurement of Colors

The strength of colors, expressed as K/S values, was evaluated by using the Kubelka-Munk equation by determining the light reflectance technique performed on an UltraScan PRO D65 UV/VIS spectrophotometer.

K/S = [(1 − R)^2^/R] − [(1 − R_o_)^2^/2R_o_] (1)

### 2.3. Fastness Properties

The fastness properties of the dyed samples like perspiration, washing, light, and rubbing were tested according to the tests of the American Association of Textile Chemists and Colorists [14].

#### 2.3.1. Washing Fastness

The composite examples were sewn between two bits of dyed cotton and wool fabrics and afterward drenched in an aqueous solution containing 5 g/L of nonionic detergents at 60 °C for 30 min. Samples were removed and dried. Assessment of the wash fastness was set up utilizing the grey scale for color change.

#### 2.3.2. Rubbing Fastness

##### Dry Rubbing 

The test specimen was placed flat on the base of the crock meter. A white testing fabric was mounted. A covered finger was lowered onto the test specimen and slid back and forth 20 times. The white test sample was then removed for evaluation using the grey scale for staining.

##### Wet Rubbing 

The white test sample was thoroughly (65%) wet with water. The procedure was run as before. The white test samples were air dried before evaluation.

#### 2.3.3. Perspiration Fastness

The acidic solution was prepared by dissolving L-histidine monohydrochloride monohydrate (0.5 g), sodium chloride (5 g), and sodium dihydrogen orthophosphate dihydrate (2.2 g) in one liter of water, and the pH was adjusted to 5.5. On the other hand, the alkaline solution was prepared by dissolving L-histidine monohydrochloride monohydrate (0.5 g), sodium chloride (5 g), and disodium hydrogen orthophosphate dihydrate (2.5 g) in one liter of water, and the pH was adjusted to 8. The colored specimen was sewn between two pieces of uncolored specimens. The composite samples were immersed for 30 min in both solutions. The test specimens were placed between two plates of glass under a force of 5 kg in an oven at 37 ± 2 °C for 4 h. The effect on the color of the tested specimens was expressed and defined by reference to the grey scale color change.

#### 2.3.4. Light Fastness

This test was carried out by utilizing a carbon arc lamp and continuous light for 35 h. The effect on the color of the tested samples was recorded through reference to the blue scale for color change.

### 2.4. Evaluation of Cytotoxicity, Antioxidant and Antimicrobial Activities

The cytotoxicity, antioxidant, and antimicrobial activities were determined at the Regional Center for Mycology and Biotechnology (RCMB) at Al-Azhar University, Cairo, Egypt.

#### 2.4.1. Evaluation of Cytotoxic Effects of Certain Chemical Compound 

Mammalian cell lines: MCF-7 cells (human breast cancer cell line), HepG-2 cells (human Hepatocellular carcinoma), HCT-116 (colon carcinoma), and A-549 cells (Lung carcinoma cell line). were obtained from the VACSERA Tissue Culture Unit. Chemicals used: dimethyl sulfoxide (DMSO), crystal violet, and trypan blue dye were purchased from Sigma (St. Louis, MO, USA). Fetal bovine serum, DMEM, RPMI-1640, HEPES buffer solution, L-glutamine, gentamycin, and 0.25% Trypsin-EDTA were purchased from Lonza (Morristown, NJ, USA). The published method was followed when evaluating anticancer activities [15].

#### 2.4.2. DPPH Radical Scavenging Activity

The methanol solution of the 2,2-diphenyl-1-picrylhydrazyl (DPPH) radical was prepared and stored at 10°C. A methanol solution of the test disperse dyes were prepared. A 40 µL aliquot of the methanol solution was added to 3mL of DPPH solution. The decrease in absorbance at 515 nm was determined continuously, with data recorded at (1 min) intervals until the absorbance stabilized (16 min). The absorbance of the DPPH radical in the absence of the antioxidant (control) and the reference compound ascorbic acid was also conducted. The percentage inhibition (PI) of the DPPH radical was calculated according to the formula:

PI = [{(*A*C − *A*T)/*A*C} × 100] (2)

where *A*C represents the absorbance of the control at t = 0 min and *A*T represents the absorbance of the sample + DPPH at t = 16 min [16]. The IC_50_ (50% inhibitory concentration), the concentration required to inhibit DPPH radical by 50%, was calculated from graphic plots of the dose response curve.

#### 2.4.3. Antimicrobial Activity Test

The antimicrobial activities of dyed fabrics with disperse dyes 1 and 2 were tested using the agar-well diffusion technique against ten different microbial cultures. Pure cultures of *Streptococcus mutants RCMB 017* (1)ATCC 25175, *Micrococcus* sp.RCMB 028(1),and *Enterococcus faecalis* (ATCC 29212), (Gram-positive bacterium), *Escherichia coli* (RCMB 010052) ATCC 25955, *Enterobacter cloacae* RCMB 001 (1) ATCC 23355 and *Proteus vulgaris* RCMB 004 (1) ATCC 13315 (Gram-negative bacterium), and *Candida albicans* RCMB 005003 (1) ATCC 10231 (fungi) were used in the test, and the published method was followed when evaluating antimicrobial activities [17].

## 3. Results and Discussion

**Figure 1:**
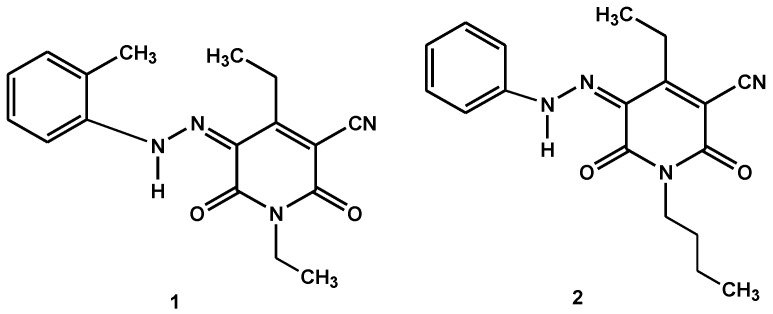
Structures of disperse dyes 1 and 2.

1,4-diethyl-2,6-dioxo-5-(o-tolyl-hydrazono)-1,2,5,6-tetrahydro-pyridine-3-carbonitrile disperse dye 1 and 1-butyl-4-ethyl-2,6-dioxo-5-(phenyl-hydrazono)-1,2,5,6-tetrahydro-pyridine-3-carbonitrile disperse dye 2 recently synthesized by one of us [18] (Figure 1) were used for dyeing polyethylene terephthalate fabrics at a shading of 2%, by using commercial HC commercial carrier as a non-eco-friendly carrier [6] and Tanavol EP 20017 as an eco-friendly carrier at dyeing temperatures of 100 °C. Greenish-yellow color shades were obtained.

### 3.1. Color Assessment

The colored fabrics were surveyed using a tristimulus colorimeter. The CIELAB (Color space defined by the International Commission on Illumination (CIE) in 1976) psychometric coordinates L*, a*, and b* represent the color hues and were estimated for the color of the dyed example, where a* represents the red–green axis; b* represents the yellow–blue axis; L* represents lightness; (c*) represents the chroma; and (h*) represents the hue angle from 0 to 360°. It is obvious from the results recorded in Table 1 that positive L* values showed that the disperse dyes were lighter and may contain a heterocyclic ring that increases the values of the L* coordinate. This truth is maintained by the estimations of c*, which were positive, while the K/S worth increments as an electron donating methyl appears, as in disperse dye 1.

The data in Table 1 demonstrates that completion of the dyeing process using the eco-friendly carrier gave better K/S results (4.74 and 3.46) than the non-eco-friendly carrier (4.04 and 3.01) at 100 °C.

### 3.2. Fastness Properties

Using disperse dyes 1 and 2 for dyeing the polyester fabrics showed excellent results for fastness to washing, rubbing, and perspiration when using both non and eco-friendly carriers, but the fastness results of these dyes to light for the dyed polyester fabrics ranged from very good to good. Improvement to the light fastness property to the dyed polyester fabrics by using zinc oxide nanoparticles is under investigation (Table 2 and Table 3).

### 3.3. In Vitro Antimicrobial Activity

The dyed polyester fabrics of disperse dyes 1 and 2 weretested for their inhibitory effects on the growth of three pathogenic Gram-negative bacterial strains *Escherichia coli* (RCMB 010052) ATCC 25955, *Enterobacter cloacae* RCMB 001 (1) ATCC 23355, and *Proteus vulgaris* RCMB 004 (1) ATCC 13315; three pathogenic Gram-positive bacterial strains *Streptococcus mutants* RCMB 017 (1) ATCC 25175, *Micrococcus sp*. RCMB 028 (1), and *Enterococcus faecalis* (ATCC 29212); one pathogenic fungi *Candida albicans* RCMB 005003 (1) ATCC 10231;gentamycin served as the standard of Gram bacteria (positive and negative), and ketoconazole served as the standard of fungi. The antimicrobial screening data revealed that the dyed polyester fabrics with disperse dye 1 or disperse dye 2 did not possess antimicrobial properties against all of the tested organisms. Functional textiles include imparting functions like antimicrobial, wash durability, and topographical function [18]. Imparting antibacterial function to the dyed polyester fabrics by using zinc oxide nanoparticles isalso under investigation.

### 3.4. In Vitro Cytotoxicity Screening

**Figure 2:**
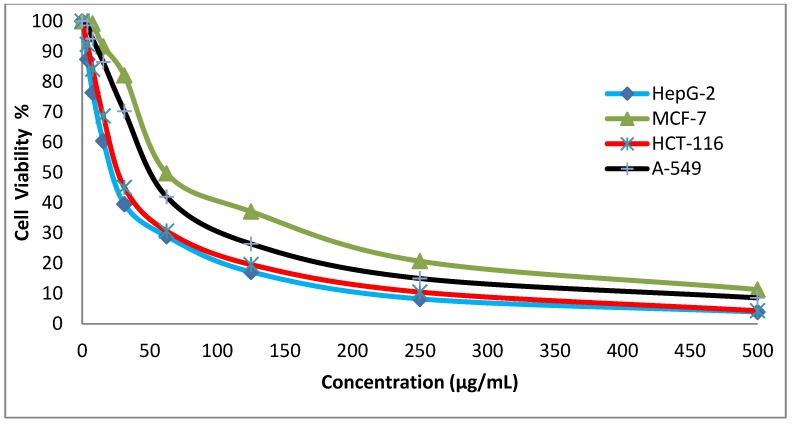
Evaluation of the cytotoxic effects of disperse dye 1. HepG-2: Hepatocellular carcinoma cells, MCF-7: breast cancer cells; HCT-116: colon carcinoma cells; A-549: Lung carcinoma cells.

**Figure 3:**
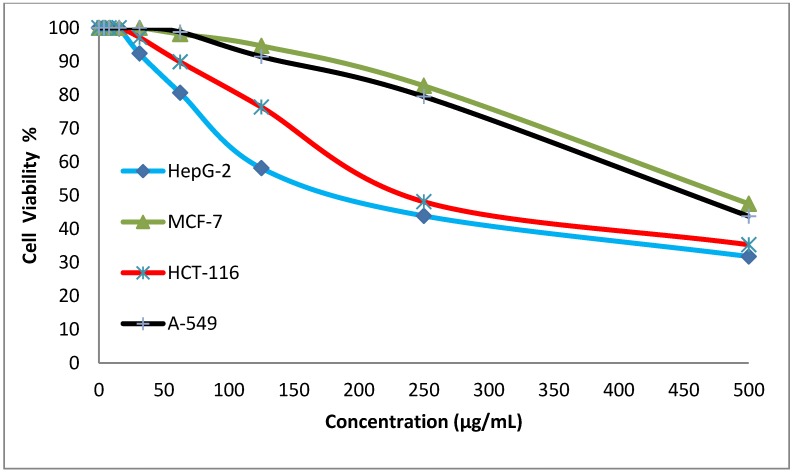
Evaluation of the cytotoxic effects of disperse dye 2.

Cytotoxicity is one of the most significant markers for biological assessment in vitro studies. In vitro, chemicals have diverse cytotoxicity mechanisms, for example, the inhibition of protein synthesis or irreversible binding to receptors [19]. The preliminary anticancer activity study of the synthesized disperse dyes 1 and 2 was evaluated against four human cell lines: HepG-2 cells (Hepatocellular carcinoma), MCF-7 cells (breast cancer), HCT-116 (colon carcinoma), and A-549 cells (Lung carcinoma) using Cisplatin and Imatinib as the reference drugs. Different concentrations of the two disperse dyes were used to calculate the IC_50_ values (concentration required to inhibit 50% of the culture growth when the cells were exposed to the tested disperse dyes for 48 h). Both Table 4 and Figure 2 and Figure 3 reveal that disperse dye 1 had strong activity, with IC_50_ values of 23.4, 62.2, 28, and 53.6 g/mL in HePG-2, MCF-7, HCT-116, and A-549 cells, respectively. On the other hand, disperse dye 2 showed weak activity with IC_50_ values of 196, 482, 242, and 456 g/mL in HePG-2, MCF-7, HCT-116, and A-549 cells, respectively.

### 3.5. Antioxidant Activity (DPPH Radical Scavenging Activity)

**Figure 4:**
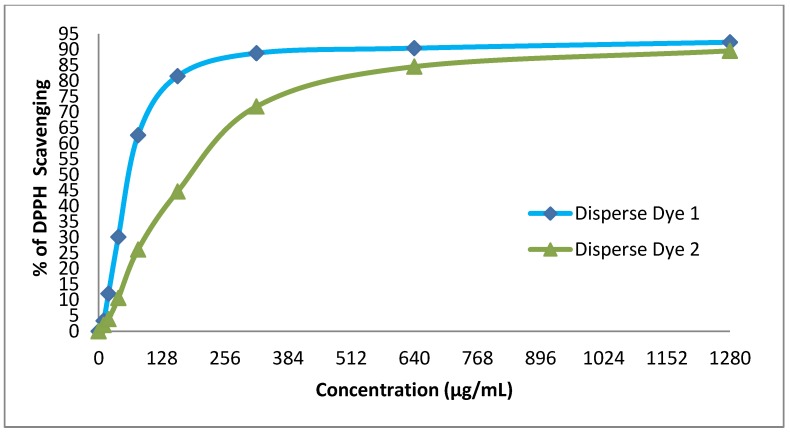
Evaluation of the antioxidant effect of disperse dyes 1 and 2.

The antioxidant property of the two disperse dyes were evaluated in vitro by the DPPH free radicals scavenging activity. The antioxidant activities of the dyes were represented in terms of IC_50_ (μg/mL concentration required to inhibit DPPH radical formation by 50%). Data listed in Table 4 reveal that the moderate antioxidant activity of disperse dye 1with an IC_50_ of 64.5 was more than the ascorbic acid as the standard with an IC_50_ of 14.2, while disperse dye 2 exhibited a weak antioxidant activity of IC_50_191.6 (Figure 4). 

## 4. Conclusions

We used disperse dyes in the dyeing of polyester fabrics at 100 °C and had excellent results for fastness to perspiration, rubbing, and washing, but the light fastness was only good. Disperse dyes 1 and 2 showed strong to weak values for both anticancer and antioxidant activities, respectively.

## Figures and Tables

**Table 1:** Optical measurements of the disperse dyes on polyester fabrics.

Dyes Number	K/S	λ_max_	L*	a*	b*	c*	h*
Eco-friendly carrier							
Disperse dye 1	4.74	445	80.06	−4.58	68.35	68.51	93.83
Disperse dye 2	3.46	450	80.35	−5.20	61.82	62.04	94.80
Non Eco-friendly carrier							
Disperse dye 1	4.04	445	80.16	−4.69	66.93	67.09	94.01
Disperse dye 2	3.01	445	80.39	−5.55	62.05	62.30	95.11

K is a measure of light absorption; S is a measure of light scattering; L* represents lightness; c* represents the chroma; and h* represents the hue angle.

**Table 2:** Fastness properties of the polyethylene terephthalate fabrics dyed with disperse dyes 1 and 2 by utilizing the eco-friendly carrier.

Dye Number	Fastness to Rubbing		Wash Fastness			Fastness to Perspiration						Fastness to Light
						Acidic			Alkaline			
	Dry	Wet	Alt	SC	SW	Alt	SC	SW	Alt	SC	SW	
Disperse dye 1	5	5	5	5	5	5	5	5	5	5	5	3–4
Disperse dye 2	5	5	5	5	5	5	5	5	5	5	5	3

where SW = Staining on wool, SC = Staining on cotton, Alt = Alteration.

**Table 3:** Fastness properties of the polyethylene terephthalate fabrics dyed with disperse dyes 1 and 2 by utilizing a non-eco-friendly carrier.

Dye Number	Fastness to Rubbing		Wash Fastness			Fastness to Perspiration						Fastness to Light
						Acidic			Alkaline			
	Dry	Wet	Alt	SC	SW	Alt	SC	SW	Alt	SC	SW	
Disperse dye 1	5	5	5	5	5	5	5	5	5	5	5	4–5
Disperse dye 2	5	5	5	5	5	5	5	5	5	5	5	4

where SW = Staining on wool, SC = Staining on cotton, Alt = Alteration.

**Table 4:** Antitumor and antioxidant activities of disperse dyes 1 and 2.

Dye Number	Cytotoxic Activity (IC_50_ µg/mL)				Antioxidant Activity (IC_50_ µg/mL)
	HepG-2	MCF-7	HCT-116	A-549	
Disperse dye 1	23.4 ± 1.2	62.2 ± 4.1	28 ± 1.9	53.6 ± 5.8	64.5
Disperse dye 2	196 ± 3.2	482 ± 8.9	242 ± 3.6	456 ± 7.3	191.6
Cisplatin	18.4 ± 0.9			19.3 ± 0.8	
Imatinib		24.6	9.7		
Ascorbic acid					14.2

HepG-2: Hepatocellular carcinoma cells, MCF-7: breast cancer cells; HCT-116: colon carcinoma cells; A-549: Lung carcinoma cells.
